# Evaluation of a Ground Subsidence Zone in an Urban Area Using Geophysical Methods

**DOI:** 10.3390/s24123757

**Published:** 2024-06-09

**Authors:** Lara De Giorgi, Dora Francesca Barbolla, Chiara Torre, Stefano Settembrini, Giovanni Leucci

**Affiliations:** 1 Institute of Heritage Science, National Research Council, Prv.le Lecce-Monteroni c/o Campus Universitario Ecotekne, 73100 Lecce, Italy; lara.degiorgi@cnr.it (L.D.G.); dora.barbolla@ispc.cnr.it (D.F.B.); 2 Dipartimento di Scienze Umane Piazza Dante, 32 University of Catania, 95124 Catania, Italy; chiara.torre@phd.unict.it; 3 Independent Researcher, Via Pio XII n. 27, 73049 Ruffano, Italy; info@geostudisettembrini.it

**Keywords:** GPR, ERT, urban area, ground subsidence risk, limestone cavity

## Abstract

An important geological risk to which many towns in Puglia are exposed is sinking cavities in urban areas. For urban centers, studying, mapping, providing geological and speleological descriptions, classifying, and cataloging the forms and types of cavities is essential because cavities are linked to past local anthropic and natural processes at different sites. These circumstances could lead to the enhancement of existing underground cavities in urban areas through conservation and continuous monitoring. Unfortunately, in many cases, these underground cavities have been used as landfills and subsequently abandoned. In late March 2007, one of these cavities collapsed inside Gallipoli’s inhabited center, causing damage to the structures but fortunately not human lives. In the area surrounding the collapsed cavity, a series of geophysical investigations were undertaken using ground penetrating radar in an attempt to delimit the area of collapse and develop possible interventions for restoration. In the same area, these measures were repeated 16 years later in December 2022 due to another collapse. The comparison between data acquired in these two periods shows that there were no strong changes apart from an increased presence of subsoil moisture in 2022.

## 1. Introduction

**Figure 1:**
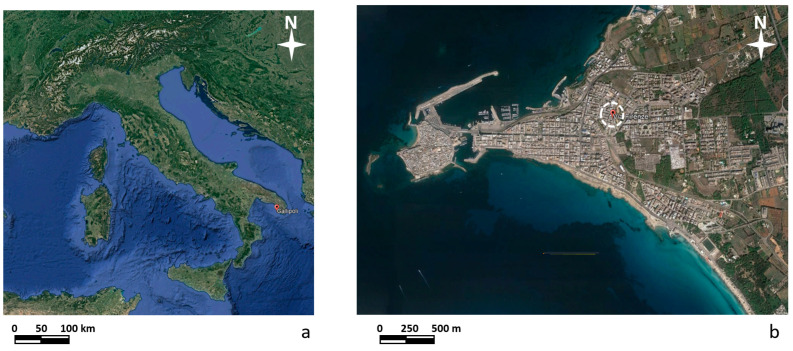
(**a**) Map of Italy indicating the location of Gallipoli; (**b**) map of Gallipoli with the location of the investigated area.

Due to the subsidence and collapse of natural and anthropic cavities, Puglia is one of the Italian regions most affected by soil subsidence [1,2]. Such events sometimes destroy or threaten infrastructure integrity, even those of particular importance, such as roads and railway lines [3]. The recurrence of instability phenomena characterizes Salento. This high frequency is linked to the area’s geology and especially to the evolution of karst processes and human activities that exploit subsoil resources and settlements in the area. In particular, the city of Gallipoli is located on the Ionian coast of Salento (Figure 1). The eastern part of Gallipoli was affected from the end of the 1800s to the beginning of the 1900s by intense underground mining activity due to calcarenite deposits. These deposits, having good mechanical properties, were used to produce tuff ashlars and as ornamental stones for external surfaces [2,3].

**Figure 2:**
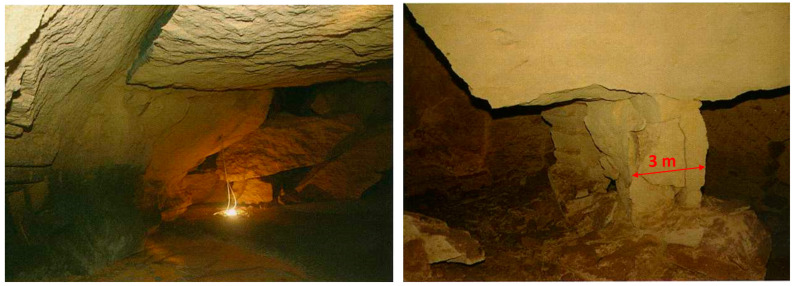
The internal part of the calcarenite quarry.

**Figure 3:**
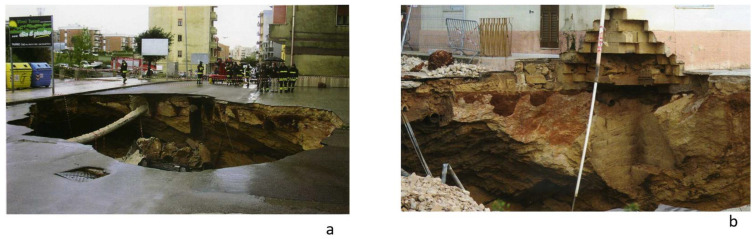
Collapse phenomena in late March 2007. (**a**) and (**b**) show the collapsed road surface in 2007.

The discovery depth of the calcarenite deposit varies between 2 m and 25 m. Mining activity developed through large halls with randomly arranged square pillars with sides equal to 2 or 3 m (Figure 2). The deposit was reached through vertical wells, which were commonly referred to as bell-shaped. Following exploitation, the quarries were abandoned and often used as waste deposits. Relative access to the deposit was blocked and buried until it fell out of memory. By the end of March 2007, a major roadway collapse event occurred (Figure 3) [2,3].

The road surface showed deformation a few days before the collapse, as indicated by the slippage of a maintenance hole cover placed on the roadway. Cracks were also detected on buildings affected by instability caused by street level subsidence. The sinking was preceded by days of more or less intense and prolonged rain. The collapse saw the formation of a chasm 12 m in diameter and 8 m deep, which involved two buildings that housed 16 families. The chasm was accompanied by fractures in the vaults and the detachment of large blocks and suspended boulders. To restore safety to unsafe buildings and prevent new collapses, geophysical investigations in the area affected by the collapse were necessary to identify any other danger zones and safe routes for the transit of mechanical intervention. Given the urgency of this first intervention, ground penetrating radar (GPR) was used.

**Figure 4:**
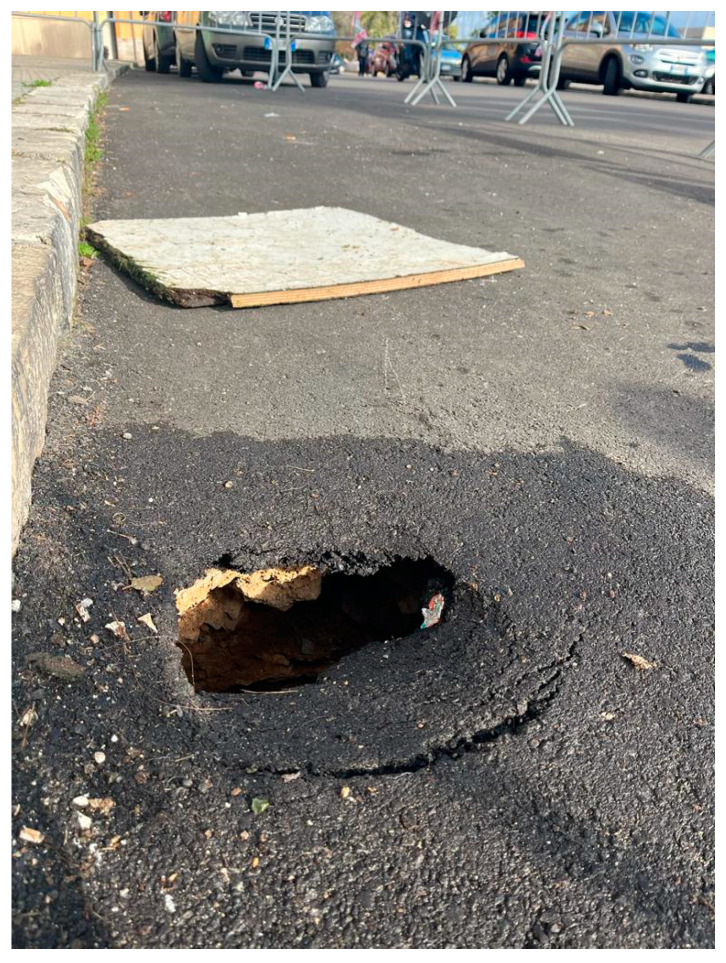
Collapse phenomena in late December 2022.

In December 2022, twenty-two years after instability phenomena in the same area, there was a slight sinking of the road pavement (Figure 4). For this reason, further geophysical investigations were necessary to verify the extent of the phenomenon. In this case, electrical resistivity tomography (ERT) and GPR were used.

International scientific literature has reported many geophysical applications to areas at risk of subsidence [4,5,6,7,8,9,10,11,12,13,14].

For example, reference [6] used electrical resistivity imaging or tomography (RESTOM) as a tool for detecting and mapping known sinkholes in dolomitic areas. This technique discriminates between developing sinkholes and mature sinkholes comprising resistive air-filled cavities. Reference [8] used ERT and GPR methods to map subsoil in karstic areas. They concluded that ERT is effective for detecting soil–rock interfaces and even irregular terrain and fracture structures, such as funnel-shaped dolines. Since soil and rock demonstrate higher resistivity and contrast, GPR can detect most fractures at different depths at study sites. The authors of [12] used 3D GPR data to detect cavities in urban areas. The authors developed a car-mounted 3D GPR system with two antenna arrays oriented in different polarization directions and detected more than 100 cavities in three Chinese cities. In this study, we integrated two geophysical methods (ERT and GPR) and compared GPR data acquired in two different campaigns (2007 and 2022).

The risk was often linked to natural cavities formed over time in karst environments. In the case of this paper, the cavities are known because they were manmade and abandoned. These cavities have posed safety issues over the years.

The geophysical investigation in this case study helped identify areas not at risk, facilitating restoration and safety measures. Fifteen years after the first instability event, geophysical investigations have shown that the risk is still present. Therefore, consolidation interventions carried out in 2007 must be redesigned.

## 2. Materials and Methods

**Figure 5:**
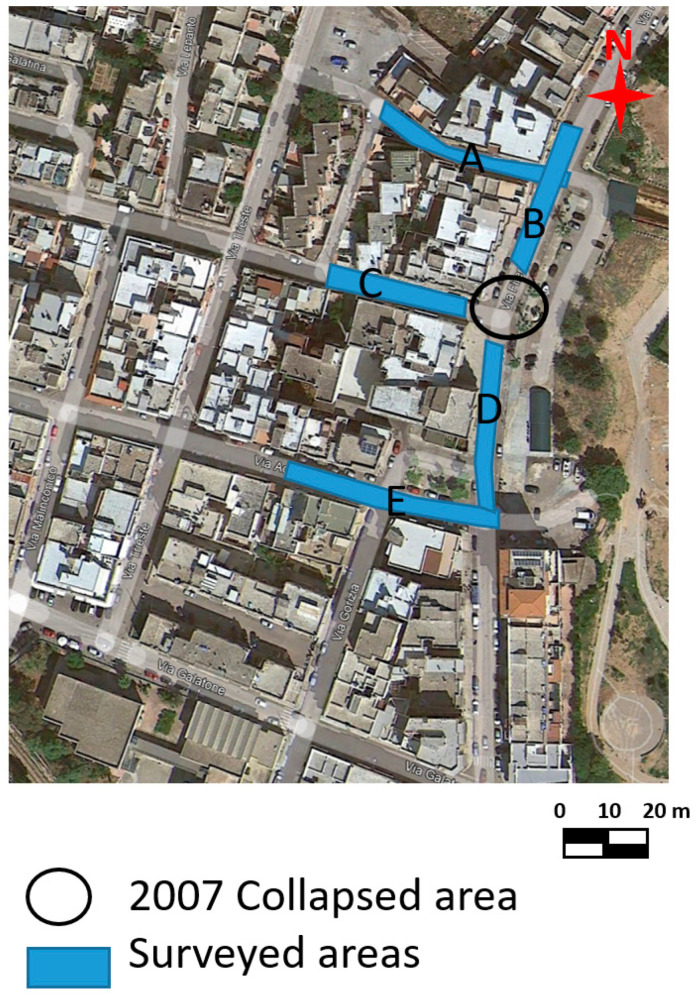
GPR surveyed areas in 2007.

For the first geophysical surveys in 2007, the georadar of Mala Geoscience with a 250 MHz antenna was used. GPR measurements were performed in areas A, B, C, D, and E (Figure 5).

GPR data were acquired in a grid with parallel profiles spaced 0.5 m.

The two-way time window was 100 ns (nanoseconds). The sample and trace intervals were 512 samples per scan and 0.02 m, respectively. GPR data were processed using GPR-Slice Software (GPR-SLICE Software 7.0, gpr-survey.com, accessed on 20 December 2014)). The 2D processing steps were as follows: (i) bandpass filter to eliminate both low- and high-frequency noise components; (ii) manual adjustment of the grain to improve visibility of deep reflection events; (iii) background removal filter to eliminate the horizontal components of the signal present on radar sections due to ringing; (iv) Kirchhoff migration using an average velocity value of electromagnetic waves equal to 0.108 m/ns.

Kirchhoff migration is based on an integral solution to the scalar wave equation. The Kirchhoff integral represents a field at a given point as a superposition of waves propagating from adjacent points in time. It provides objects that reflect EM waves at their hypothetical real size.

**Figure 6:**
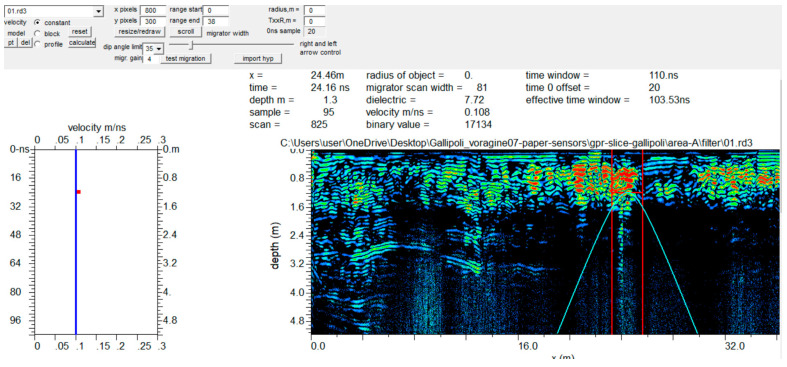
EM wave velocity analysis on a 2D radar section. The estimated EM velocity was 0.108 m/ns.

Electromagnetic (EM) wave velocity can be estimated from GPR data in several ways. The conventional method involves common depth points (CDPs) and wide-angle reflection and refraction (WARR) datasets. Both methods require two antennas in separate units and relatively long acquisition times [9,10]. The EM wave velocity can be more quickly and easily determined from the reflection profiles acquired in continuous mode, using the characteristic hyperbolic shape of reflection from a point source (diffraction hyperbola) [9,10,11,12]. This method is common for EM velocity estimation and is based on the phenomenon that a small object (the object dimensions are smaller than the wavelength of the EM wave introduced into the ground) reflects EM waves in almost every direction. Figure 6 shows an example of how the above method was applied to the data acquired in the investigated areas.

The processed data were subsequently used to construct 3D volumes. In this way, the data can be viewed in various ways, which simplifies their interpretation.

**Figure 7:**
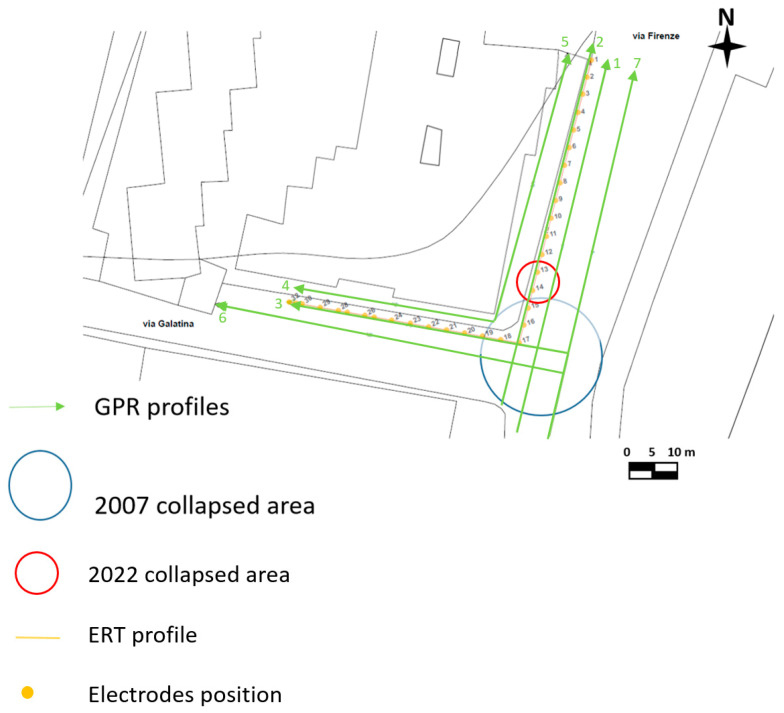
GPR and ERT surveyed areas in 2022.

For the second geophysical survey of 2022, the georadar Ris Hi-mod with a dual-band antenna of 200–600 MHz was used. Figure 7 shows the location of GPR profiles. GPR data were processed using GPR-Slice software (GPR-SLICE software 7.0 (gpr-survey.com, accessed on 20 December 2014) with the same processing steps used for data acquired in 2007.

**Figure 8:**
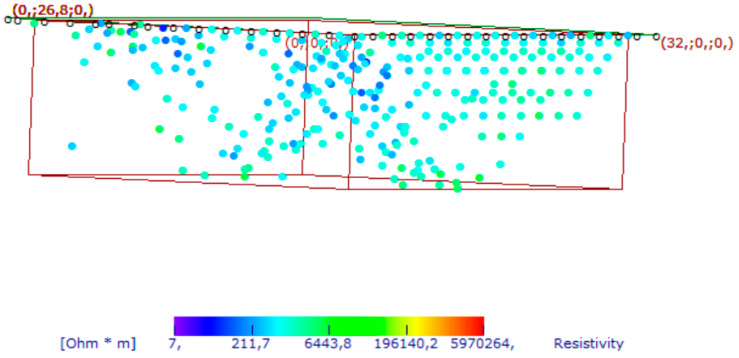
Apparent resistivity of measured points. The white circles represent electrode locations.

For the second geophysical survey in 2022, ERT data were acquired with an MAE 600 E georesistivimeter with 32 active channels. In this case, the survey was performed using a particular geometry (L shape) to investigate below the building [13,14,15,16]. The L profile is shown in Figure 7. The dipole–dipole array was considered as it is commonly used for cavity detection [13,14,15,16]. The distance between the electrodes was equal to 1 m, and 32 electrodes were used. This type of array is well-described in the international literature [14,15,16]. Initially, a 2D survey is conducted of each perpendicular line or transect. In the next step, the current electrodes remain at the end of one line while the potential electrodes are positioned along the line. Then, the current electrodes move one electrode position, and the potential electrodes move as previously described. This process is repeated until the current and potential electrodes cover the L geometry. This sequence of observations produces apparent resistivity toward and beneath the central portion of the array. The colored circles in Figure 8 represent the attribution points, where the apparent resistivities are measured, for the performed ERT array. This process is discussed in detail by [16].

Electrocardiogram electrodes were used to avoid piercing the floor [17,18,19,20]. The contact resistance was low (about 2000–3000 Ω m), probably due to high humidity in road paving.

ERTLab software (http://www.geostudiastier.it, accessed on 20 December 2014) was used to process the data in 3D mode. The software uses the tetrahedral finite element iterative method (data variance iterative reweighting).

## 3. Results

### 3.1. The 2007 GPR Data Analysis

#### 3.1.1. Area A

**Figure 9:**
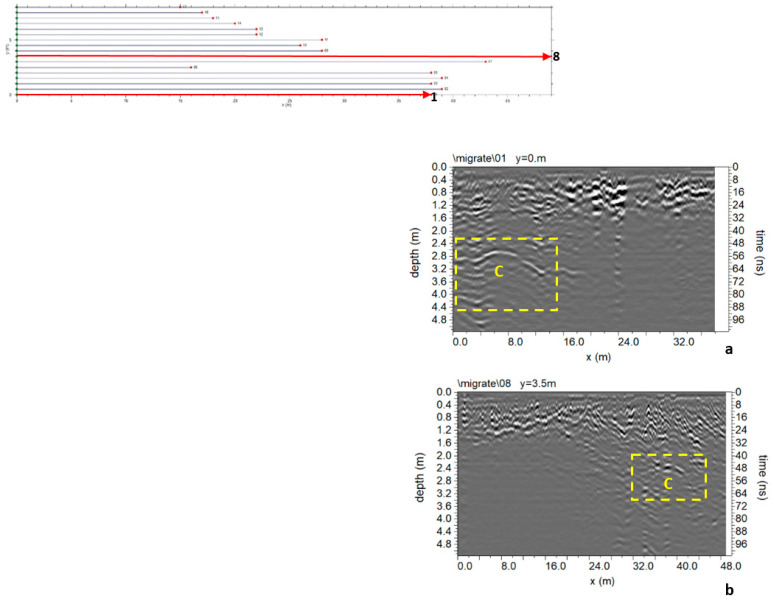
Area A: GPR processed data: (**a**) profile n. 1; (**b**) profile n. 8; C indicates a probable cavity.

In Area A (Figure 5), 17 parallel profiles spaced 0.5 m apart were acquired. The processed data (Figure 9) present several reflection events, including a subsoil with backfill material in the first meter of depth. An interesting reflection event denoted as “C” in Figure 9a is evident in the first four profiles acquired in area A. The size is about 15 m wide, and the top depth is between 2.6 and 3.2 m (with an average electromagnetic wave velocity of 0.108 m/ns).

Another interesting reflection event is “C” (Figure 9b). The size is about 8 m wide, and the top depth is between 2.4 and 2.8 m. Some considerations can be made to understand what may be causing C. When these reflection events occur in the radar sections, the polarity of the electromagnetic wave (EM) changes. A polarity inversion is produced when the reflection coefficient is negative [10,11]. Therefore, for materials where wave velocity depends only on dielectric permittivity, radar waves are reflected in materials with higher dielectric permittivity. A typical case involves reflections from air to any other material [10,11]. Based on these considerations, cavities are interpreted as the cause of these anomalies.

A 3D map of the distribution of anomalies identified within 2D radar sections was built. One visualization type is related to the construction of 2D time slices at specific time intervals [10,11]. In this work, time slices were built to visualize amplitude variations within time intervals Δt = 10.4 ns. Overlay analysis was also used [19,20,21,22].

**Figure 10:**
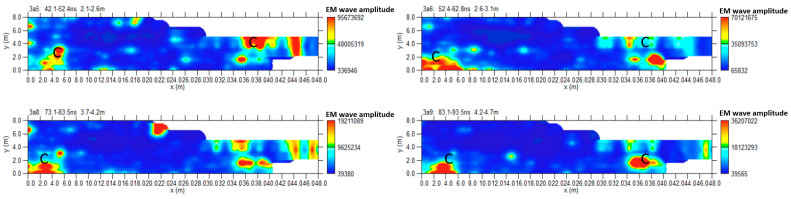
Area A: GPR time slices; C indicates a probable cavity.

Figure 10 shows the most significant time slices. Slices at depths between 2.1 m and 4.7 m are considered. At these depths, it is possible to highlight the reflected event linked to C anomalies corresponding to the one indicated by C in the 2D radar section of Figure 9.

Another way to present 3D data volume is the iso-surface amplitude of the EM wave (Leucci, Conyers). The 3D structure of anomalies identified in the 2D radar sections can be uniquely identified by isolating the amplitude values and establishing a minimum threshold value. The threshold is very delicate and depends heavily on the interpreter’s experience to obtain valuable results [10,11].

**Figure 11:**
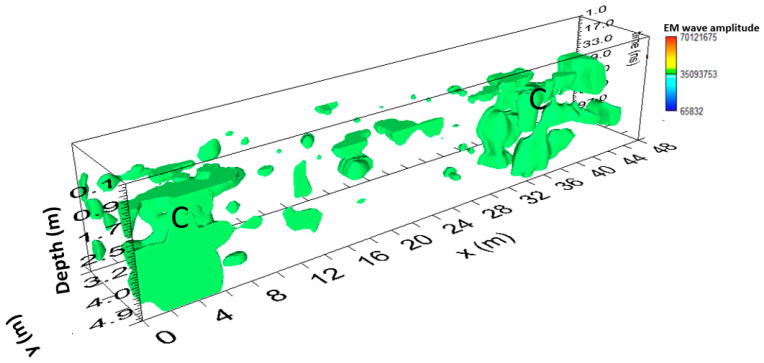
Area A: GPR iso-surfaces; C indicates a probable cavity.

Figure 11 shows the iso-surface amplitude with a threshold value equal to 65% of the total amplitude. C anomalies can be visualized in a 3D environment.

#### 3.1.2. Area B

**Figure 12:**
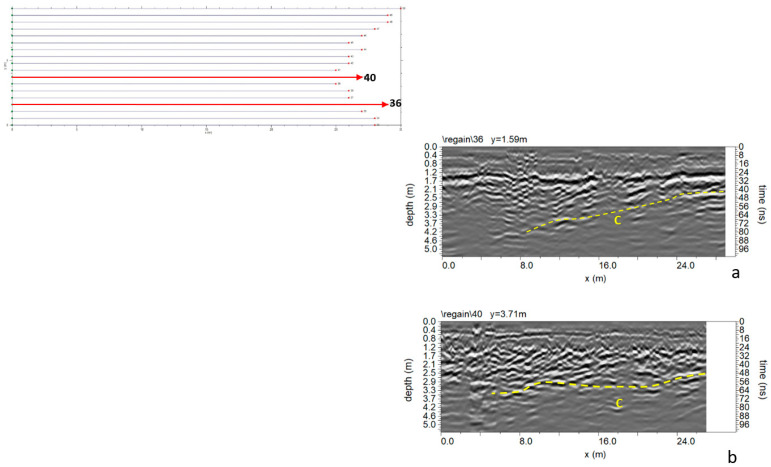
Area B: GPR processed data: (**a**) profile n. 36; (**b**) profile n. 40; C indicates a probable cavity.

In Area B (Figure 5), 18 parallel profiles 0.5 m spaced were acquired. At the end of Area B is the collapsed part of the road surface. The processed data shows an interesting reflection event “C” in Figure 12, which appears along the entire radar section at a depth between 2.0 m and 4.0 m. Since a polarity inversion is noted, this anomaly was probably due to cavities.

**Figure 13:**
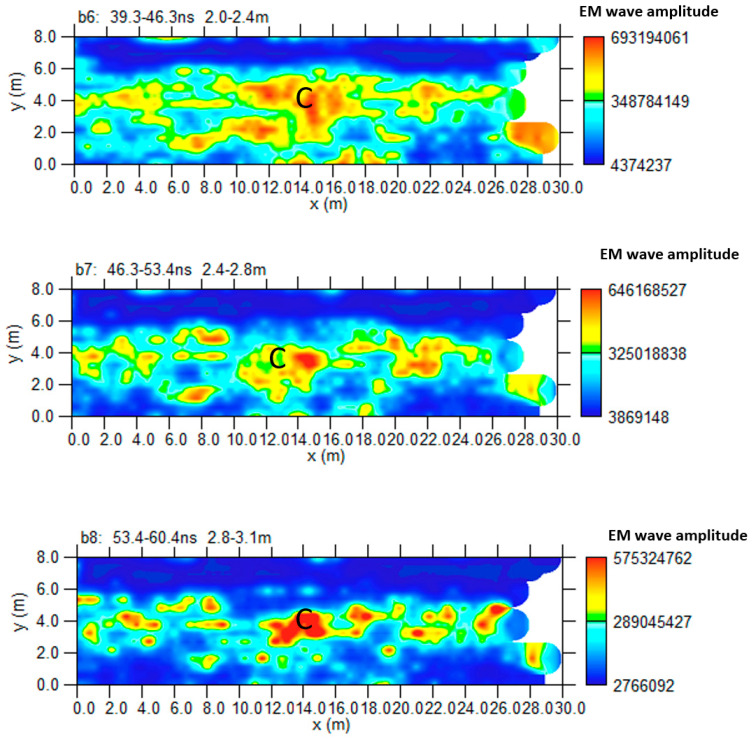
Area B: GPR time slices; C indicates a probable cavity.

Figure 13 shows the most significant time slices. Slices at depths between 2.0 m and 3.1 m are considered. It is possible to highlight the reflected event linked to anomaly C at these depths.

**Figure 14:**
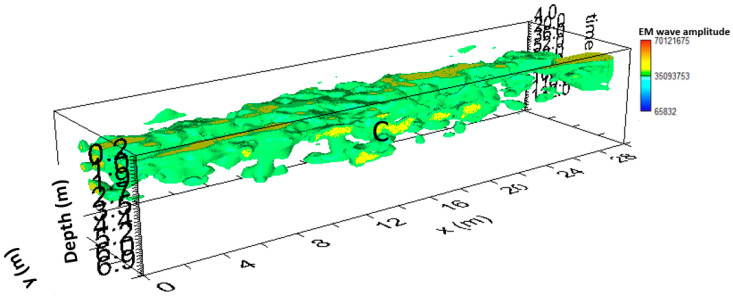
Area B: GPR iso-surfaces; C indicates a probable cavity.

Figure 14 shows the iso-surface amplitude with a threshold value equal to 70% of the total amplitude. Anomaly C can be visualized in a 3D environment.

#### 3.1.3. Area C

**Figure 15:**
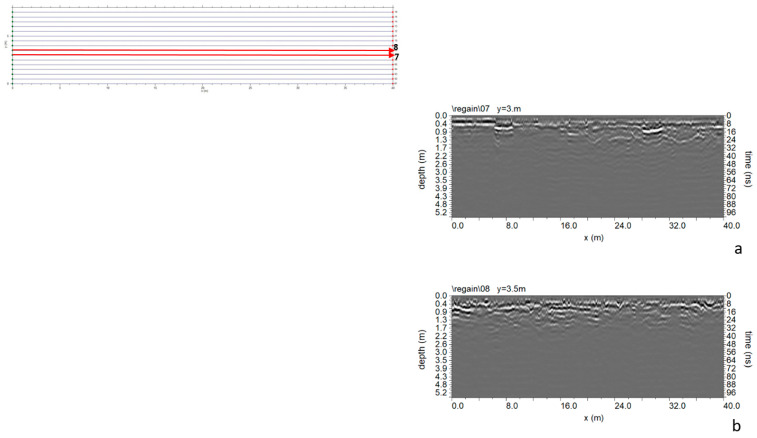
Area C: GPR processed data: (**a**) profile n. 7; (**b**) profile n. 8.

In Area C (Figure 5), 16 parallel profiles spaced 0.5 m apart were acquired. The GPR data analysis did not reveal any anomalies attributed to the presence of cavities (Figure 15).

#### 3.1.4. Area D

**Figure 16:**
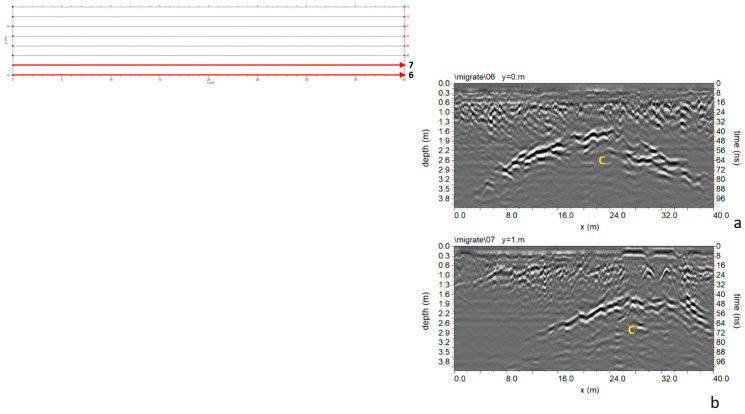
Area D: GPR processed data: (**a**) profile n. 6; (**b**) profile n. 7.

**Figure 17:**
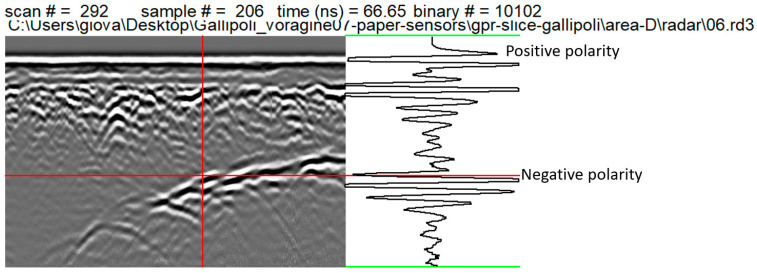
Area D: polarity inversion in the reflection event.

In Area D (Figure 5), eight parallel profiles spaced 1.0 m apart were acquired. The processed data shows an interesting reflection event “C” in Figure 16. It develops at a depth between 1.6 m and 3.0 m. The polarity is inverted (Figure 17), indicating cavities may be responsible for this anomaly.

**Figure 18:**
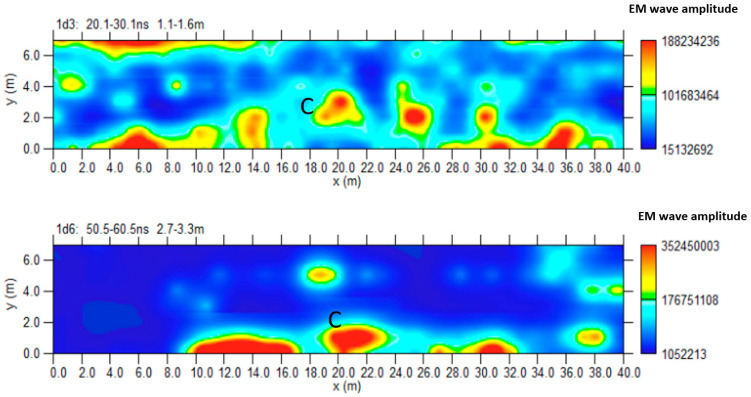
Area D: GPR time slices; C indicates a probable cavity.

Figure 18 shows the most significant time slices. Slices at depths between 1.1 m and 3.3 m are considered. It is possible to highlight the reflected event linked to anomaly C at these depths.

**Figure 19:**
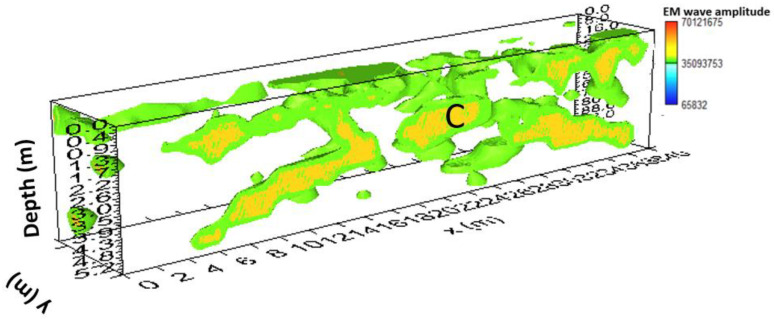
Area D: GPR iso-surfaces; C indicates a probable cavity.

Figure 19 shows the iso-surface amplitude with a threshold value equal to 70% of the total amplitude. Anomaly C can be visualized in a 3D environment.

#### 3.1.5. Area E

**Figure 20:**
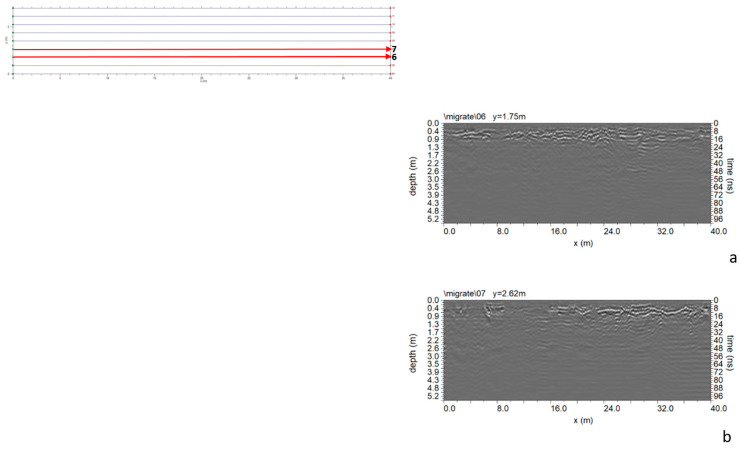
Area E: GPR processed data: (**a**) profile n. 6; (**b**) profile n. 7.

In Area E (Figure 5), nine parallel profiles spaced 0.85 m apart were acquired. The processed data did not reveal any anomalies attributed to the presence of cavities (Figure 20).

### 3.2. The 2022 GPR Data Analysis

**Figure 21:**
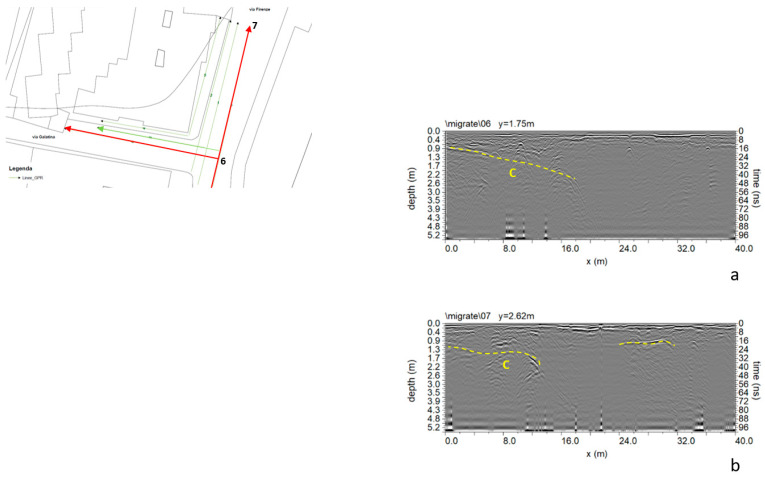
GPR processed data: (**a**) profile n. 6; (**b**) profile n. 7; C indicates a probable cavity.

The second subsidence event that occurred 15 years after the first made a second campaign of measures necessary. In this case, some GPR profiles were performed to understand the phenomenon’s extent. Therefore, investigations were carried out only in the area affected by the second instability event. From the analysis of the data (Figure 21), it is possible to notice reflected events “C”, which confirms the presence of a cavity. A polarity inversion of the reflected EM wave can also be observed [10,11,20].

### 3.3. The 2022 ERT Data Analysis

**Figure 22:**
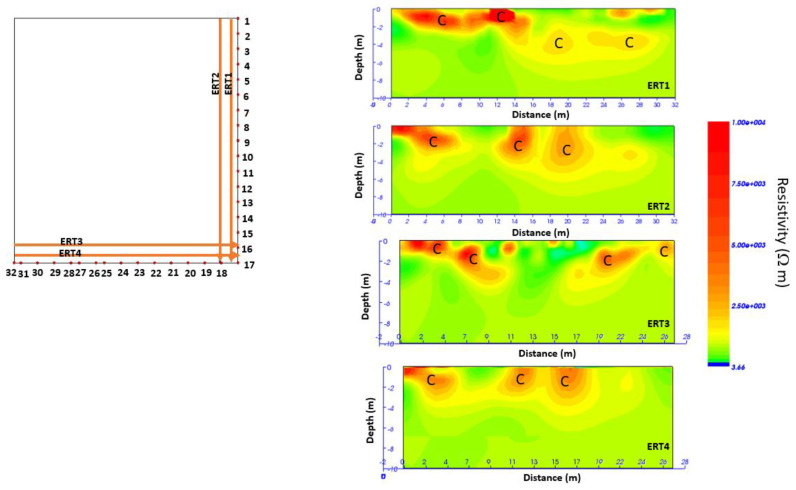
Two-dimensional resistivity distribution; C indicates a probable cavity.

The ERT investigation was necessary to understand the development of the cavity beneath the buildings. For this reason, the acquisition geometry shown in Figure 7 was adopted. Figure 22 shows the 2D distribution of electrical resistivity as a function of depth.

From the 2D resistivity distribution (Figure 22), the presence of a heterogeneous subsoil with resistivity values between 2000 Ω m and 10,000 Ω m is evident. In particular, the presence of areas (indicated by C) with resistivity values between 2500 Ω m and 10,000 Ω m suggests the probable presence of cavities. These cavities seem to extend to a depth of about 4 m.

**Figure 23:**
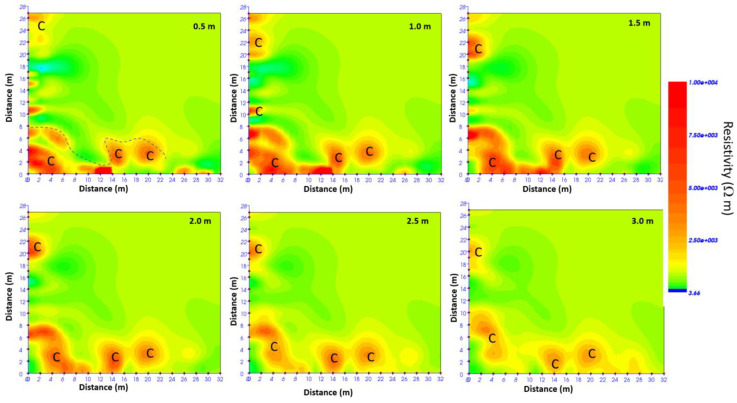
ERT depth slices; C indicates a probable cavity.

It is possible to build ERT depth slices to visualize the resistivity distribution in a 3D mode below the investigated area within depth intervals. Here, all anomalies with the same resistivity values are interpolated, and a 3D volume of resistivity data is built. It is also possible to extract the 2D resistivity distribution at several depths. Figure 23 shows the most significant time slices.

From the depth slice analysis (Figure 23) the distribution of the resistivity anomalies “C” is below the investigated area. 

**Figure 24:**
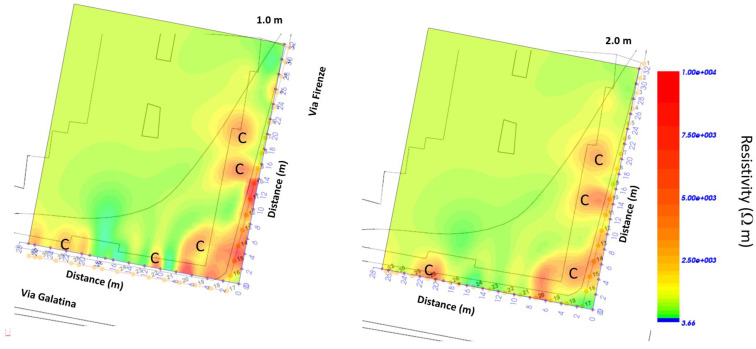
ERT depth slices overlapped to the planimetry; C indicates a probable cavity.

Figure 24 shows the superimposition of the ERT results on the plan of the investigated area showing the position of probable cavity C.

Here, it is possible to see how the cavity develops in a dangerous manner beneath the buildings.

## 4. Discussion

In addition to restoring safety in the area affected by the collapse, the geophysical investigation aimed to better understand the stability conditions of unexplorable cavities in adjacent areas. A serious problem was found in areas where the cavity’s roof thickness was less than 5 m (areas A, B, and D). As shown in Figure 7, Figure 10 and Figure 14, the roof thickness varied from 2.4 m (Figure 7) and 2.9 m (Figure 9) to 1.9 m (Figure 14). In this case, buildings near these areas could be in danger. Areas C and E were the most suitable for heavy vehicles to travel through for the first consolidation works. In the collapse area, consolidation intervention involved filling the cavities with concrete and strengthening the pillars with spritz-beton (shot concrete) inside, into which a filling of loose material (stone) was inserted.

**Figure 25:**
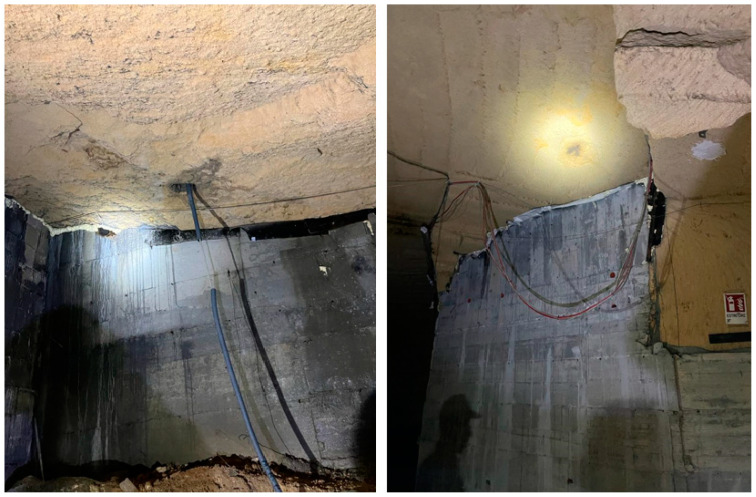
Photos of the consolidation interventions.

A cement conglomerate (also called sprayed concrete or shotcrete in English) is sprayed at high speeds toward the surface with a compressed air lance. Spritz-beton is used in consolidation interventions where concrete must be placed without formwork and high mechanical resistance must be achieved in a short period. A cement mixture with setting accelerators is created, which allows for almost instant bonding. Figure 25 shows the photos of the cavities after intervention.

**Figure 26:**
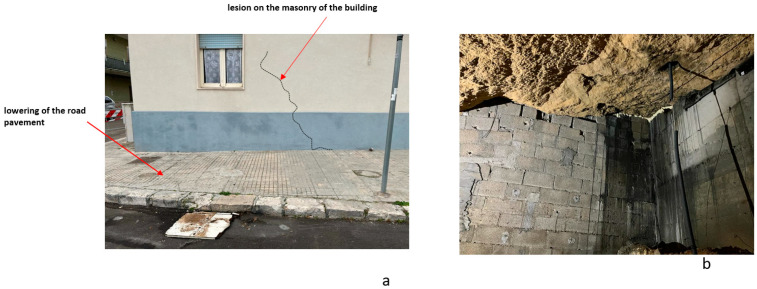
Photos: (**a**) December 2022 phenomenon; (**b**) the cavity below the building after consolidation interventions.

In December 2022 (15 years after the first collapse event), another small collapse occurred in the same area as the first collapse. There was a small hole with road pavement subsidence and cracks in the building (Figure 26a). Pavement sagging was also noticed inside the building (photos are not shown for privacy reasons). For this reason, the geophysical survey was limited to the area affected by the phenomenon. GPR results showed the presence of a cavity with roof thickness thinning ranging from 0.9 m to 1.3 m (Figure 19). The ERT results helped us understand what lies beneath the building. Already at a depth of 0.5 m (Figure 20 and Figure 21), cavities could be seen. Cavity inspection revealed a probable deterioration of the consolidation intervention (Figure 26b).

**Figure 27:**
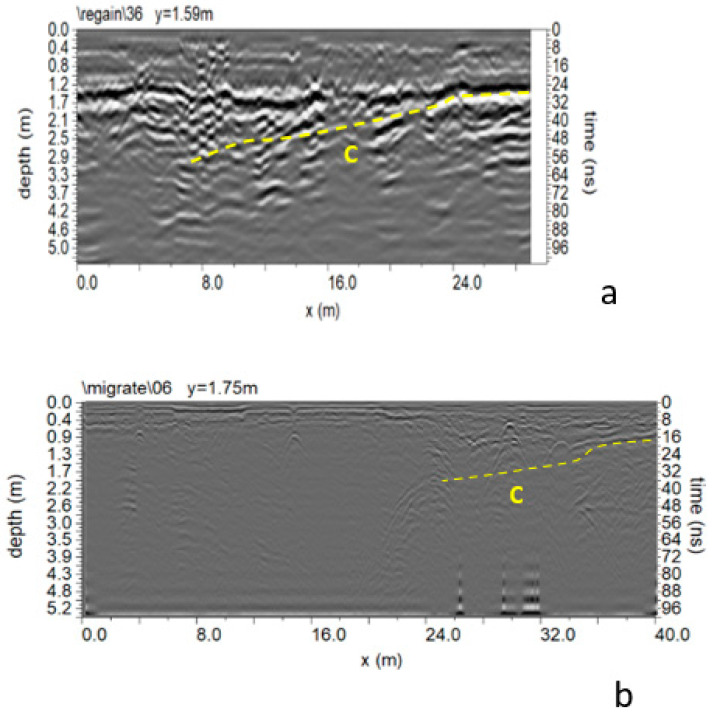
Comparison between GPR data acquired in the 2007 campaign (**a**) and GPR data acquired in the 2022 campaign (**b**).

**Figure 28:**
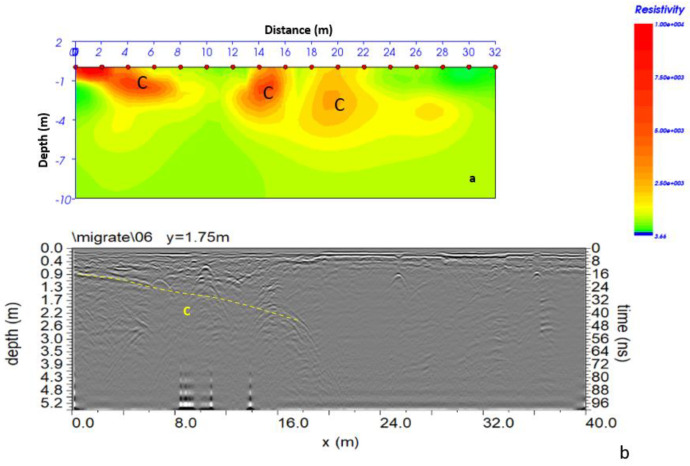
Comparison between ERT and GPR data acquired in the 2022 campaign. (**a**) ERT data; (**b**) GPR data.

Comparing the GPR data acquired in the 2007 campaign to those acquired in the 2022 campaign (Figure 27), there were no notable changes in 15 years apart from greater humidity in the subsoil, which reduced the depth of the investigation for data acquired in 2022. The comparison between ERT and GPR data acquired in the 2022 campaign was also interesting (Figure 28). The correspondence between high resistivity anomalies (C) in Figure 28a and reflection events (dashed yellow line C) in Figure 28b is easily observed. It is possible to note the polarity inversion of reflected EM waves.

## 5. Conclusions

Immediately after the event in late March 2007, geophysical investigations became necessary to understand the development of the phenomenon. They also indicated the safest routes for rescuers during the first interventions. In this case, the geophysical survey using GPR showed the cavity’s development and the safest routes for intervention. Subsequently, to make the cavity safe, a series of reinforced concrete pillars were built inside, over which a filling of loose material (stone) was inserted. Over the years, this material (due to abundant rain) likely disintegrated, causing the second instability event in December 2022. In this case, integrated geophysical surveys using ERT and GPR highlighted the presence of voids where the fill was made. Such voids are also present under houses. Furthermore, comparing GPR data acquired in two different campaigns allowed control of the cavities’ probable evolution. These findings suggested that the cavity from 2007 did not change; rather, the ERT 3D survey showed its development underneath buildings.

## Data Availability

The data presented in this study are available upon request from the corresponding author.
